# Children's peer violence perpetration and victimization: Prevalence and associated factors among school children in Afghanistan

**DOI:** 10.1371/journal.pone.0192768

**Published:** 2018-02-13

**Authors:** Julienne Corboz, Osman Hemat, Wahid Siddiq, Rachel Jewkes

**Affiliations:** 1 Gender and Health Research Unit, South African Medical Research Council, Pretoria, South Africa; 2 Help the Afghan Children, Kabul, Afghanistan; Pennsylvania State University, UNITED STATES

## Abstract

**Background:**

Child peer violence is a global problem and seriously impacts children’s physical and psychological health, and their education outcomes. There are few research studies on children’s peer violence available in South Asian countries, particularly in Afghanistan. This paper describes the prevalence of children’s peer violence perpetration and victimization and associated factors among school children in Afghanistan.

**Methods:**

A total of 770 children were recruited into a baseline study conducted as part of an intervention evaluation in 11 schools (seven girls’ and four boys’ schools). All children were interviewed with a questionnaire developed for the study. The main outcome is a three-level peer violence variable consisting of (a) no violence, (b) victimization only, or (c) perpetration (with or without victimization). Peer violence victimization was measured through the Multidimensional Peer-Victimization Scale, and peer violence perpetration was measured through an adjusted version of the same scale with wording changed to measure perpetration.

**Results:**

49.7% of boys and 43.3% of girls reported having experienced more than one instance of violence victimization in the past month, and 31.7% of boys and 17.6% of girls disclosed perpetration of more than one instance of violence in the past month, with considerable overlap found between experience of victimization and perpetration, particularly among boys. Multinomial models of factors associated with peer violence show that for boys, food insecurity was associated with perpetration of peer violence but not with victimization, and experiencing corporal punishment at school in the last month was significantly associated with both peer victimization and perpetration. For girls, food insecurity, more depressive symptoms and experiencing any beating at home were associated with both violence victimization and perpetration. Having a disability was associated with victimization only, and having witnessed their father fighting and experiencing any kind of corporal punishment were associated with peer violence perpetration only.

**Discussion:**

Peer violence in Afghanistan is linked to food insecurity, exposure of children to witnessing family violence, and children’s experience of physical violence at home and corporal punishment at school. School-based settings provide an important platform for interventions to reduce and prevent peer violence; however, such interventions may benefit from broader violence-prevention initiatives conducted at the community level.

## Introduction

Peer violence can be defined as “intentional and repeated infliction of harm on a person by one or more peers that are usually more powerful in some regard” [1]. Peer violence or victimization may be direct (physical aggression, threats and teasing) or indirect (spreading rumours and exclusion from peer groups) [2], and may involve bullying, which is generally a more targeted and chronic or repetitive type of peer violence [3].

The prevalence of experience of peer violence victimization among children in schools varies globally. Studies have suggested that 20.8% of children between the ages of 12 and 18 reported being bullied at school in the USA [4], between 11.3% and 49.8% children globally reported experiencing bullying (in predominantly highly industrialized contexts) [5], and between 7% and 59% children in South East Asian countries reported having been bullied [6]. Less is known about prevalence of peer violence in South Asian countries, with the majority of available studies comprising broader child violence studies with smaller components on child bullying or victimization [7]. One study from India reported that 60.4% of children aged 8–14 years had been bullied [8]. Data from the Global School-based Student Health Survey suggests that the prevalence of having been bullied in the past month among 13–15 year old students is 41.1% in Pakistan [9] and 44.2% in Afghanistan [10]. However, more recent studies conducted in Pakistan and Afghanistan suggest that experience of peer victimization may be much higher, with a recent study on violence against children in five provinces of Afghanistan suggesting that 63% of children had been victimized by their peers [11] and a study conducted in one province of Pakistan showing experience of peer victimization among sixth grade children as high as 90.8% for boys and 75.3% for girls [12].

Much research suggests that boys experience more peer violence than girls [7, 13], with one study implemented among adolescent school children in 40 countries indicating prevalence of any kind of involvement in peer violence (as the victim, perpetrator or both) ranging from between 8.6% and 45.2% for boys and between 4.8% and 35.8% for girls [14]. These findings are largely consistent in South Asia, with more boys than girls reporting having experienced peer victimization in Pakistan (45.1% of boys and 35.3% of girls) [9], India (63.9% of boys and 53% of girls) [8] and Bhutan (57% of boys and 47% of girls) [15]. However, the findings from Afghanistan vary slightly, where 42.3% of boys compared with 44.9% of girls reported being bullied in the past month according to the Global School-based Student Health Survey [10], with more boys than girls reporting having been beaten by other children in a smaller study [11].

The negative effects of peer violence on children’s health and psychological wellbeing have been widely documented. Experience of peer violence victimization has been linked to children’s physical health problems and psychosomatic symptoms, such as headache, fatigue, stomach pain and dizziness [16, 17], and psychological problems including poor self-esteem, feelings of depression, social anxiety, sleep disturbance, lower self-efficacy, loneliness, hopelessness and suicidal ideation [8, 16, 18, 19, 20, 21]. Peer violence may also impact negatively on children’s school performance, with some studies suggesting that experience of victimization or perpetration of peer violence are associated with poor academic performance or attendance [22, 23, 24, 25, 26].

Research has identified a number of factors associated with peer violence. A recent systematic review on predictors of peer violence perpetration found a range of individual, family and school-level factors associated with violence [27]. For instance, at the individual level, peer violence perpetration is commonly associated with a range of socio-demographic, physical, psychological and relational factors. Notably, perpetration appears to be linked to children’s violence-supportive and patriarchal gender attitudes [28, 29]. At the family level, exposure to family violence emerges as a strong predictor of peer violence perpetration [27, 30]. The findings related to family socio-economic status are mixed. A systematic review suggests that in some cases violence victimization and perpetration is more common among children from wealthier families [27], with other studies suggesting that children who are victimized or perpetrate violence are more likely to be poor [31] or food insecure [32]. Recent studies conducted in South Asia suggest that poverty and food insecurity may be strong factors associated with peer violence. In India, Pells et al. [19] found that poorer children were more likely to be bullied than wealthier children. In Pakistan, Karmaliani et al. [12] found that girls and boys who perpetrated peer violence, and girls who experienced peer violence victimization, were more food insecure than children who had not experienced any kind of peer violence.

The lack of evidence related to peer violence prevalence and associated factors in South Asia, particularly in Afghanistan, was a rationale for including an evaluation of an Afghan NGO’s school-based peace education intervention in the portfolio of interventions to be evaluated under the UK-Aid funded What Works To Prevent Violence Against Women and Girls? Global Programme. In this paper we draw on data from the baseline evaluation of this intervention. The aims of the paper are to describe the prevalence of peer violence among seventh and eighth grade school children in one province of Afghanistan and associations between socio-economic status, school performance, mental health, gender attitudes, child violence-supportive attitudes, and experiences of violence at home and corporal punishment at school.

## Methods

The data were derived from a baseline survey conducted to evaluate the intervention Preventing Violence Against Women and Girls, implemented by the Afghan NGO Help the Afghan Children (HTAC). The baseline pre-test survey will be followed up with a midline survey during the intervention and an endline survey post-intervention.

HTAC’s intervention includes school-based peace and conflict resolution education with boys and girls, complemented with a range of community activities including conflict resolution and peace building training with community and religious leaders and women’s civil society actors, and the dissemination of media messaging related to the prevention of violence against women and girls. The peace education program in schools is provided over a two-year period for a cohort of grade seven and eight students who will be graduating from grades eight and nine at the end of the intervention. Peace education classes occur outside of regular school hours (either before or after formal classes) and enrolment in the classes is voluntary for all students.

The baseline study was conducted among grade seven and eight students in 11 secondary schools (seven girls’ and four boys’ schools) receiving the intervention in three districts of Jawzjan province (Sheberghan, Aqcha and Faizabad). The original sample size was 720 students: 360 boys and 360 girls. HTAC enrolled 2000 boys and 1500 girls (a total of 3500 students) into their peace education program, and the sample size was calculated as that needed to assess if there was a trend across the study time points of a reduction in scores of peer victimization and perpetration among the children. It was anticipated that a sample size of 720 children would be adequate for this, but it was unknown what the scores would be at baseline.

In total, 770 students (350 boys and 420 girls) were randomly sampled for the baseline survey due to a small error in the data collection process that saw a slight under-sampling of boys and an oversampling of girls. Sixty girls were sampled from each girls’ school and between 82 and 91 boys were sampled from each boys’ school. Children participating in HTAC’s peace education classes and their parents were requested to provide informed consent and a random sample was obtained from the final list of children with confirmed personal and parental consent. The random selection was done prior to data collection such that enumerators were provided with the names and details of students to be sampled, with the details of additional students also provided in case selected students were not available or present at school on the day allocated for data collection.

All children were interviewed with a questionnaire designed for the study and based on a questionnaire developed in English and piloted in Pakistan for research conducted with children of a similar age (administered in Urdu and Sindhi languages) [33]. Pilots in Pakistan indicated that the survey was culturally and age appropriate for Pakistani students. The questionnaire was also piloted in Afghanistan prior to baseline data collection, in schools in Kabul province having received HTAC’s intervention in the past, with minor adjustments subsequently made to the wording and translation of questions. The instrument was written to 6^th^ grade reading level (in English) and translated into Dari. Due to low literacy levels among sixth grade Afghan children when compared with students in neighbouring countries [34], questionnaires were administered by trained enumerators with female enumerators interviewing girls and male enumerators interviewing boys. All questionnaires were entered into an SPSS data file and cleaned.

Children were asked their age, school grade, how many people lived in their home and how many siblings (brothers and sisters) they had. Socio-economic status was assessed through two questions about the frequency of going to school without breakfast and to bed without dinner. Values for these questions ranged from between zero and three (0 = never, 1 = once, 2 = two or three times, and 3 = four or more times). Values for these two questions were summed to give a food insecurity/hunger score. Students’ disability was measured through five questions derived from the Washington Group on Disability Scale: do you have difficulty seeing, even if wearing glasses, do you have difficulty hearing, do you have difficulty walking or climbing, do you have difficulty remembering or concentrating, do you have difficulty speaking. Possible response categories include no difficulty, some difficulty, a lot of difficulty or cannot do at all. Students were coded as having a disability if they responded ‘a lot of difficulty’ or ‘cannot do at all’ to any of the five questions, in line with Washington Group analytic guidelines [35].

A number of questions were asked to assess children’s experiences and performance at school, including the number of days absent from school in the past month, and the main reason for school absence for the last day missed from school (being ill, no money for transport, helping at home, working to earn money, afraid to go to school due to violence, or another reason). Students were also administered simple literacy and numeracy tests, including reading one line of the questionnaire, and completing three mathematics equations (16+4, 7+3, 25/5). For literacy, students were coded as reading fluently if they were able to read the line of the questionnaire with ease. For numeracy, students were coded as numerate if they were able to give the correct answer for the third (division) equation.

A gender attitudes scale was used with 13 items. The gender attitudes questions had a 4-point Likert response scale (strongly agree to strongly disagree), with values from zero to three, and were developed for the setting. Students were asked to respond to four statements related to women’s participation in everyday activities, including weddings, neighborhood events, skills training and income generating activities. In addition, students were asked to respond to the following nine statements: I think girls in our family should go to school; I think the husbands in my family should give permission to their wives to go to the clinic; I think the husbands in the family should listen to their wives’ opinion on schooling; I think the wives in my family should have a say in how money in their family is spent; I think the wives in my family should be able to ask a religious scholar about issues; I think the husbands in my family should respect the opinion of their wives on matters related to income generating work; I think a husband in my family should be kind and caring toward the women in his family; I think that the wives in my family should always obey their husbands; and I think that if a wife in my family does something wrong her husband has the right to punish her. Values for the last two questions were reversed and all questions were summed to give a gender (inequitable) attitudes score with a possible range of between zero and 27. Cronbach’s alpha was 0.87 for girls and 0.84 for boys.

Students’ experience of depression was measured through a modified Children’s Depression Inventory (CDI—2) [36], which includes 28 items related to children’s self-rated feelings of sadness, self-hate, negative self-image, suicidal ideation, loneliness, fatigue etc. in the last two weeks. Values range from zero to two with higher values indicating more severity of depressive symptoms, for instance: ‘I am never sad’, ‘I am sad once in a while’ and ‘I am sad many times’. Scale scores were computed by summing item responses. Scores on the total scale have a possible range of 0 to 56 with higher scores indicating more depressive symptoms. Cronbach’s alpha was 0.73 for girls and 0.83 for boys.

Students’ sense of hope was measured through a scale modified from Snyder et al.’s Hope Scale [37]. The modified scale has six items measured on a 4-point Likert response scale (strongly disagree to strongly agree), with values from zero to three: I can think of many ways to get out of a difficult situation; I put lots of energy into pursuing my goals; there are lots of ways around any problem; I can think of many ways to get the things in life that are important to me; even when others get discouraged, I know I can find a way to solve the problem; and I meet the goals that I set for myself. All items were summed to give a hope score with a possible range of between zero and 18, with higher scores indicating more hope. Cronbach’s alpha was 0.72 for girls and 0.76 for boys.

Students were asked questions about their observation and experience of violence at home. Students were asked two questions about having observed abuse of their mother in the previous month, one related to physical violence from her husband and the other about physical violence from any family member. A variable was derived as a measure of any abuse of the mother by her husband or other family member in the past month. A question was also asked if the child had seen or heard his or her father fighting physically with another man in the last month. Students were asked how often in the past month they had been slapped, hit, beaten or physically punished by a parent, and whether they had been beaten so hard at home in the past month that they were injured. A variable was derived from these two questions as a measure of any experience of violence at home in the past month.

Students’ experience of corporal punishment by teachers at school was measured through six questions about how often in the past month teachers had: slapped, hit, beaten or physically punished students, twisted students’ ears, made students stand on a bench, made students run around as punishment, made students kneel down, or hit students with a stick, whip or other object. A variable was derived from these six questions as a measure of any experience of corporal punishment at school in the past month.

An attitudes towards child punishment and fighting scale was developed for the context and contained five items. The attitudes to child punishment questions had a 4-point Likert response scale (strongly disagree to strongly agree), with values from zero to three. They required a response to the following statements: I think that if a child disobeys their parents they should be beaten; I think that if a child gets into fights their parents should beat them; I think that if a child talks back to their parents they should be punished by being beaten; I think a child who misbehaves at school should be beaten; and I think that if a child hurts me I should hurt them back. All questions were summed to give a child punishment attitudes score with a possible range of between zero and 18, with higher scores indicating more support for violent practices. Cronbach’s alpha was 0.89 for girls and 0.9 for boys.

The Peer Victimization Scale is a 16-item measure designed for young people aged 11 to 17, with 4 subscales, each with 4 questions assessing physical victimization, verbal victimization, social manipulation, and property attacks in the previous 4 weeks [38]. Students were asked over the past month how often (i.e., never, once, a few times (2–3) or many times (4 or more) an event happened to them). Scale scores were computed by summing item responses, with scores having a possible range of 0 to 48. Cronbach’s alpha was 0.87 for girls and 0.84 for boys. A Peer Perpetration Scale was developed for the study based on the peer victimization scale. The scale consisted of the same 16 peer victimization scale items with the wording adjusted to measure perpetration [38] and the same subscales and approach to scoring. Cronbach’s alpha was 0.86 for girls and 0.74 for boys.

This study was approved by the Institutional Review Board (IRB) of the Ministry of Public Health, Afghanistan, and the Ethics Committee of the South African Medical Research Council. HTAC obtained all necessary permissions from the Provincial Education Directorate in Jawzjan province and schools participating in the study. HTAC provided a verbal description of the study to school principals, teachers and students, and consent forms and information sheets were translated into Dari and given to children to take home and give to their parents. Only children with signed consent forms from their parents and who gave verbal consent themselves were recruited into the study.

## Data analysis

The data was analyzed using Stata 13.0 with students clustered into schools. Variables were summarized as percentages or means, and the statistical test of difference between categories was a Pearson chi square test for categorical variables and t-tests for continuous variables. The main outcome used in the analysis was a three-level peer violence variable consisting of no violence (victimization or perpetration), victimization only, and perpetration (with or without victimization). The 16 victimization and 16 perpetration questions were scored by adding the item values, and new victimization and perpetration variables were created based on the CDC definition of peer violence which differentiates between no/low violence (if there has been zero or one type or episode of violence) and victimization or perpetration of violence (if there has been more than one episode or type of violence) [39]. A three level variable was then derived with levels referred to as ‘no/low violence’ (n = 148 boys and 223 girls), ‘victimization only’ (n = 91 boys and n = 123 girls) and ‘perpetration (with or without victimization)’ (n = 111 boys and n = 74 girls). In the bivariable analysis, for both categorical and continuous variables, two p values are presented: no violence compared with victimization only, and no violence compared with violence perpetration (with or without victimization).

In order to determine factors associated with victimization and perpetration among girls and boys, multinomial regression models were built with the three level peer violence variable as the outcome. Independent variables were the social and demographic characteristics of the children, disability, school attendance and literacy/numeracy variables, violence at home, corporal punishment at school, having observed their mother being abused, having observed their father fighting, depression and gender attitudes. The variables were entered into the model and sequential backwards elimination was used, removing those with the largest value for p first until the final variables were retained at p< or = 0.05.

## Results

The baseline survey was conducted with 770 children from 11 schools (seven girls and four boys), with 420 girls and 350 boys sampled. 49.7% of boys and 43.3% of girls reported having experienced more than one instance of violence victimization in the past month, and 31.7% of boys and 17.6% of girls disclosed perpetration of more than one instance of violence in the past month. There was considerable overlap between experience of victimization and perpetration, particularly among boys. 41.7% of boys reported both victimization and perpetration, 7.1% only perpetration, 8% only victimization and 43.1% neither. In contrast, 14% of girls reported both victimization and perpetration, 30% only perpetration, 29.3% only victimization, and 26.7% had neither victimized nor perpetrated.

Table 1 shows the social and demographic characteristics of children by disclosure of perpetration and victimization, disaggregated by gender. The mean age of both boys and girls ranged between 14 and 15 years, with victimized boys and girls being significantly younger than their peers who had neither been victimized nor had perpetrated (p = 0.041 for boys and p = 0.05 for girls). There were no significant differences in age between children who had not engaged in violence and those who had perpetrated, for either boys or girls. Although there were no significant differences between peer violence group, gender and number of household members, girls who perpetrated violence had significantly more brothers than girls who reported no victimization or perpetration (p = 0.013). No other differences were found between peer violence, gender and number of siblings. Slightly fewer boys than girls reported having gone to school without breakfast in the past month (10.3% of boys and 14.8% of girls) or going to bed without dinner (8% of boys and 8.6% of girls). There were significant differences in hunger/food security, a measure of socio-economic status, by peer victimization and perpetration. Boys and girls who reported having experienced victimization or having perpetrated peer violence scored significantly higher on the hunger scale than those boys and girls who reported no violence.

**Table 1:** Social and demographic characteristics of girls and boys in Afghanistan by experience of peer violence.

	Boys							Girls						
		Neither		Victimization only		Perpetration (+/-victimization)			Neither		Victimization only		Perpetration (+/-victimization)	
Social Demographics	n	mean (SD)	n	mean (SD)	n	mean (SD)	p value	n	mean (SD)	n	mean (SD)	n	mean (SD)	p value
Age	148	14.79(1.23)	91	14.2(1.25)	111	14.29(1.26)	0.041	223	14.3(1.38)	123	14.06(1.2)	74	14.34(1.33)	0.05
							0.073							0.815
# Family members	148	9.34(3.42)	91	9.71(3.78)	111	9.58(3.26)	0.544	221	8.97(2.44)	123	9.02(2.76)	74	8.86(2.23)	0.877
							0.642							0.652
# Brothers	148	3.59(1.59)	91	3.95(1.51)	111	3.8(1.64)	0.298	222	2.97(1.55)	123	2.91(1.61)	74	3.41(1.55)	0.688
							0.423							0.013
# Sisters	148	3.18(1.86)	91	2.82(1.43)	111	3(1.83)	0.29	222	3.64(1.65)	123	3.42(1.57)	74	3.42(1.42)	0.221
							0.302							0.379
Hunger score	148	0.04(0.23)	91	0.29(0.56)	111	0.42(0.87)	0.043	223	0.10(0.39)	123	0.64(1.29)	74	1.01(1.85)	0.005
							0.018							0.004

Table 2 shows the school performance characteristics of students by disclosure of perpetration and victimization, disaggregated by gender. Overall, boys missed school in the past month for a mean of 2.44 days (range 0–20, SD = 2.27) and girls missed school a mean of 2.24 days (range 0–19, SD = 2.25). Among boys, school absence was significantly greater for those who perpetrated violence than those who experienced no violence (p = 0.047) (no significant effect was found for victimization). In contrast, for girls, school absence was significantly greater for those who experienced victimization than those reporting no violence (p = 0.013) (no significant effect was found for perpetration). For both boys and girls, there were no significant differences in numeracy according to whether they had been a victim or perpetrator of violence. Although there was no significant difference in literacy by peer violence category for boys, a significantly higher proportion of girls who had perpetrated violence were literate (i.e. read fluently) when compared with their peers who had no experience of violence (p = 0.009).

**Table 2:** School performance characteristics by experience of peer violence.

	Boys							Girls						
		Neither		Victimization only		Perpetration (+/-victimization)			Neither		Victimization only		Perpetration (+/-victimization)	
School characteristics	n	%	n	%	n	%	p value	n	%	n	%	n	%	p value
School grade: grade 7	70	47.3	49	53.85	53	47.75	0.69	108	48.43	68	55.28	34	45.95	0.42
School grade: grade 8	78	52.7	42	46.15	58	52.25		115	51.57	55	44.72	40	54.05	
# days absent from school last month (mean(SD))	148	2.37(2.04)	91	2.41(2.63)	111	2.56(2.26)	0.897 0.047	223	2(1.86)	123	2.68(2.97)	74	2.22(1.84)	0.013 0.630
Last day off as ill	27	18.24	20	21.98	30	27.03	0.422	77	34.53	47	38.21	3	40.54	0.345
Last day off helping at home	42	28.38	30	32.97	32	28.83	0.455	95	42.6	54	43.9	34	45.95	0.739
Last day off working to earn money	20	13.51	6	6.59	7	6.31	0.161	3	1.35	1	0.81	0	0	0.535
Reading: Cannot read	16	10.81	9	9.89	12	10.81	0.64	22	9.87	12	9.76	3	4.05	0.05
Reading: Reads with a little difficulty	44	29.73	30	32.97	43	38.74		43	19.28	24	19.51	6	8.11	
Reading: Reads fluently	88	59.46	52	57.14	56	50.45		158	70.85	87	70.73	65	87.84	
Maths: Not numerate at all	27	18.24	21	23.08	24	21.62	0.67	65	29.15	38	30.89	17	22.97	0.42
Maths: Adds with ease	71	47.97	47	51.65	56	50.45		50	22.42	19	15.45	10	13.51	
Maths: Divides with ease	50	33.78	23	25.27	31	27.93		108	48.43	66	53.66	47	63.51	

Overall, few children reported having observed their mother being abused by their father or another relative in the past month (3.4% of boys and 6.7% of girls) or their father hitting another man in the past month (5.1% of boys and 14.5% of girls). Nevertheless, boys and girls who had perpetrated violence were significantly more likely to have witnessed abuse of their mother, and girls who had been victimized and who perpetrated were significantly more likely to have seen their father fight with another man (Table 3). Both boys and girls who had been victimized and who had perpetrated violence were also significantly more likely to have experienced any physical violence at home, particularly girls. Boys who had been victimized and who had perpetrated violence were significantly more likely to have experienced corporal punishment at school; however, for girls, corporal punishment was only significantly associated with perpetration of peer violence. There was no significant difference in attitudes towards child punishment by peer violence category for either boys or girls.

**Table 3:** Violence at home and at school by experience of peer violence.

	Boys							Girls						
		Neither		Victimization only		Perpetration (+/-victimization)			Neither		Victimization only		Perpetration (+/-victimization)	
Violence	n	%	n	%	n	%	p value	n	%	n	%	n	%	p value
Any abuse of mother	1	0.68	3	3.30	8	7.21	0.121	7	3.14	9	7.32	12	16.22	0.237
							0.023							0.002
Father physical fight	6	4.05	6	8.79	4	3.60	0.167	7	3.14	22	17.89	32	43.24	0.013
							0.899							0.0005
Any physical violence at home	9	6.08	18	19.78	31	27.93	0.026	8	3.59	39	31.71	37	50.00	0.001
							0.044							0.0002
Any corporal punishment at school	18	12.16	54	59.34	83	74.77	0.029	49	21.97	50	40.65	49	66.22	0.129
							0.009							0.002
Attitudes to child punishment (mean (SD))	148	5.73(3.53)	91	5.43(3.48)	111	7.27(3.87)	0.784	223	3.37(3.26)	123	2.27(2.51)	74	2.62(2.79)	0.212
							0.279							0.375

No differences were found in (patriarchal) gender attitudes by peer violence category for either boys or girls (Table 4). However, these results may be linked to the different items included in the gender attitudes scale. Very large proportions of children overall, regardless of engagement in peer violence, agreed with most of the gender supportive statements included in the scale (e.g. in relation to girls’ access to education, women’s access to health, and women’s participation in income generating activities). However, large proportions of children who perpetrated violence (65.76%) agreed that a husband had the right to punish his wife if she did something wrong, compared with 54.68% of victimized children and 34.5% of children who had not experienced any peer violence. For boys, no differences were found in disability, depression or hope by peer victimization or perpetration; however, girls who had been victimized were significantly more likely to be disabled than girls who had no experience of violence (Table 4). Girls who had been victimized and who had perpetrated violence were also more likely to report symptoms of depression than girls reporting no violence. Interestingly, girls who had been victimized have significantly higher sense of hope than girls who had not experienced any violence (p = 0.03); however, the difference in mean scores is small (13.12 for no violence and 13.89 for victimization) and so this finding should be read with caution.

**Table 4:** Disability, gender attitudes and psychological wellbeing by experience of peer violence.

	Boys							Girls						
		Neither		Victimization only		Perpetration (+/-victimization)			Neither		Victimization only		Perpetration (+/-victimization)	
	n	%	n	%	n	%	p value	n	%	n	%	n	%	p value
Disability	15	10.14	13	14.29	17	15.32	0.373	36	16.14	41	33.33	16	21.62	0.0007
							0.359							0.242
Patriarchal gender attitudes scale (mean (SD))	148	11.92(3.42)	91	10.67(3.94)	110	12.18(3.83)	0.322	223	11.12(3.46)	123	9.45(3.95)	74	8.46(4.39)	0.106
							0.771							0.078
Depression CDI-2 score (mean (SD))	148	11.69(7.36)	91	14.26(6.92)	111	16.41(6.74)	0.280	223	10.20(5.45)	123	14.67(4.93)	74	15.69(5.74)	0.001
							0.121							0.004
Hope score (mean (SD))	148	14.05(2.33)	91	14.59(2.22)	110	14.19(2.08)	0.530	223	13.12(1.98)	123	13.89(2.02)	74	13.80(1.92)	0.030
							0.831							0.131

The multinomial models of factors associated with peer violence are shown in Table 5; for each model the comparison category is no violence engagement. For boys, age was associated with peer victimization but not perpetration, and being hungry was associated with perpetration of peer violence but not with victimization. Having experienced any corporal punishment in the last month was significantly associated with both peer victimization and perpetration. For girls, more hunger, being able to read fluently, more depressive symptoms and experiencing any beating at home were associated with both violence victimization and perpetration. Younger age and having a disability were both associated with victimization only, and having witnessed their father fighting and experiencing any kind of corporal punishment were associated with peer violence perpetration only.

**Table 5:** Multinomial regression models of factors associated with girls’ and boys’ experiences of peer violence.

	Victimization vs no violence				Perpetration vs no violence			
**Boys**	**RRR**	**95% confidence interval**		**p value**	**RRR**	**95% confidence interval**		**p value**
Age	0.73	0.62	0.86	0.009	0.80	0.52	1.22	0.189
Hunger score	3.68	0.81	16.82	0.072	4.53	1.58	12.98	0.02
Any corporal punishment	8.81	1.37	56.82	0.034	17.59	4.09	75.70	0.008
**Girls**								
Age	0.86	0.82	0.90	0.000	1.05	0.88	1.25	0.543
Hunger score	1.57	1.00	2.47	0.05	1.75	1.18	2.61	0.014
Reads fluently	1.51	1.09	2.11	0.022	4.27	1.07	16.98	0.042
Disability	2.08	1.03	4.20	0.044	1.33	0.34	5.17	0.625
Witnessed father fighting with a man	1.85	0.45	7.63	0.331	4.60	1.27	16.58	0.027
Depression score (CDI-2)	1.12	1.01	1.25	0.035	1.14	1.02	1.29	0.029
Any beating at home	4.73	1.35	16.53	0.023	4.57	1.68	12.43	0.01
Any corporal punishment	1.49	0.50	4.40	0.406	3.84	1.16	12.74	0.033

## Discussion

Peer violence is common among boys and girls in schools in Jawzjan province of Afghanistan, particularly experiences of peer victimization among boys, with prevalence figures comparable with peer violence in other South Asian studies, particularly in Pakistan and Afghanistan [9, 10], with the current study indicating higher prevalence for girls than in other South Asian contexts. In contrast to data from the Global School-based Student Health Survey [10], this study found higher prevalence of peer violence among boys when compared with girls in Afghanistan, in line with global patterns [13]. Although there is substantial overlap between peer victimization and perpetration among boys in this study, the overlap is smaller for girls with higher proportions of girls than boys either only experiencing victimization or only perpetrating violence against their peers.

This study has found strong associations between children’s victimization and perpetration of peer violence and exposure to violence at home, either through experiencing physical punishment or witnessing violence between adults. The study also suggests that children who had been victimized and who had perpetrated violence in the past month were significantly more likely to have experienced any corporal punishment by teachers at school. These findings are in line with other studies that highlight the importance of exposure to family violence in the perpetration of peer violence [27, 30], and a link between children’s experiences of school-based corporal punishment and perpetration of peer violence [40, 41]. As a consequence, although peer violence prevention interventions working with children are important, they should be complemented with interventions that target broader violence-supportive norms and practices at the household, community and school levels. More awareness raising needs to be conducted with families in relation to the multigenerational nature of violence, and how children learn violent norms within their households and reproduce violent behaviours in their school environments. Interventions also need to address teachers’ use of corporal punishment in schools. In a study conducted by Save the Children in Afghanistan, the majority of teachers interviewed believed that the physical punishment of students was an unavoidable form of maintaining classroom discipline [42]. Interventions thus need to focus on building teachers’ and community members’ awareness of the negative impact of school-based corporal punishment, building the capacity of teachers to reduce corporal punishment and adopt non-violent forms of discipline, and implementing appropriate child protection systems at the school level [42].

A link between peer violence and poverty has not been found in all settings [27], but a meta-analysis of bullying literature does suggest that children who are victims of bullying are more likely to have low socio-economic status, although the same study found a weak association between bullying perpetration and low socioeconomic status [43]. A recent study conducted in the USA found that food insecurity was significantly associated with school children’s experience of peer violence victimization and bullying perpetration [44]. The present study suggests that in Afghanistan there is a strong relationship between poverty (measured through hunger or food insecurity) and peer victimization and perpetration, for both boys and girls but particularly the latter. Findings related to food insecurity are in line with other studies in South Asia, which show a link between peer victimization and hunger in India [19] and Pakistan [12, 45], and peer violence perpetration and hunger in Pakistan [12].

The link between poverty and peer violence is likely complex and multilayered. The broader global literature suggests that the link between children’s peer violence victimization and structural inequalities such as poverty may be a result of discrimination against children from poorer households [19, 43]. The association between peer violence perpetration and poverty may also be partially explained by structural inequalities that lead perpetrators to seek social domination over their peers and increase their access to peer acceptance and resources [43]. The literature also suggests that there is a link between low socio-economic status and more adverse home environments, including experience of and exposure to violence [43]. More specifically in relation to food insecurity, a study conducted in the USA suggests that childhood hunger is linked to interpersonal violence and impulse control later in adulthood, and that this may be due to the negative impact of hunger on childhood neocortical development [46].

Given the multilayered nature of the association between food insecurity and peer violence, a multilayered intervention approach is likely required. Government schools should consider providing meals to students, an activity that governmental and non-governmental organizations have implemented in other South Asian Countries, such as India, with some evidence that meal provision programs in schools can improve children’s school enrolment [47] and attendance [48]. Given the strong link found in this study between food insecurity and violence perpetration and victimization, school-based meal provision may also assist in reducing children’s engagement in peer violence. However, such interventions may not be sufficient on their own and should be complemented with childhood education on antidiscrimination and respect for diversity. Interventions targeting violence against children and between adults at the household level may also mitigate the relationship between poverty and children’s experience of peer violence at school.

Peer violence is associated with students’ school performance; however, the directionality of the finding is unexpected. Although numeracy ability was not related to any kind of violence victimization or perpetration for either boys or girls, a significantly higher proportion of girls who had perpetrated violence were literate when compared with their peers who had no experience of violence, and this effect was retained in the multinomial model. This is in contrast to studies that suggest that peer violence is linked to poorer academic performance [23, 34, 25]. It is possible that a key factor is the type of violence perpetration, with several studies suggesting that perpetration of indirect or relational bullying (rather than direct and physically violent bullying) may be associated with higher academic achievement among some children [49, 50]. Woods and Wolke [50] argue that the association between relational bullying and higher academic achievement could be due to relational bullies being socially skilled at relational manipulation and more skilled in not being caught by avoiding more direct, physical bullying behaviours.

Although some research suggests that patriarchal (gender inequitable) attitudes and violence supportive attitudes are associated with more involvement in peer violence [28, 29], the current study found no significant associations between children’s gender inequitable attitudes or child violence supportive attitudes and engagement in peer violence. It is possible that in a culturally conservative and conflict-affected setting such as Afghanistan, where everyday violence against children is normalized [51], violence-supportive attitudes cease to interact with peer violence perpetration. Although the same argument could be stated for gender inequitable attitudes, recent data suggests that patriarchal attitudes in Afghanistan vary widely according to the types of rights that should be afforded to women and girls [52]. The results of this study suggest that although, overall, gender attitudes may not interact with engagement in peer violence, specific attitudes related to violence against women (i.e. men having the right to punish their wives) are associated with children’s peer violence victimization and perpetration, particularly the latter.

In line with the global literature linking peer violence with depression, girls who had been victimized and who had perpetrated violence were more likely to report symptoms of depression than girls reporting no violence, although no corresponding effects were found for boys. Furthermore, girls who had been victimized were significantly more likely to be disabled than girls who perpetrated or who had no experience of violence. Although a strong link has been made between peer victimization and disability in industrialized countries [53], less is known about the effects of peer victimization on disabled children in less industrialized settings. In Afghanistan, where people with disabilities, including children, are discriminated against and highly excluded from education, health services and other opportunities [54], but where little is known about the vulnerability of disabled children to various forms of violence, conducting research on the connections between disability and peer violence would contribute to an important gap in the literature.

## Conclusion

Thus study has shown that much like in other global contexts, including in other South Asian countries, peer violence among boys and girls in 7^th^ and 8^th^ grades in Jawzjan province of Afghanistan is highly prevalent. This study shows that children’s food insecurity, observation of family violence at home, and experience of physical violence at home and corporal punishment at school, are particularly associated with peer violence victimization and/or perpetration. The prevention of violence in Afghanistan is a highly challenging objective given extremely high rates of prevalence of interpersonal violence across society and the normalization of everyday violence, partially a result of decades of war and civil conflict. The findings of this study suggest that in Afghanistan, the prevention of peer violence should adopt a multilayered approach that implements interventions at the household, community and school levels. HTAC’s intervention model in Jawzjan province, which complements children’s peace education with broader training and awareness raising in peaceful and non-violent conflict resolution among community members, may be an important approach to reducing children’s experience of violence both at school and at home. Our research will assess how HTAC’s programming contributes to this objective.

## Supporting information

S1 File This file contains data collected from 770 students. (DTA)

S2 File This file contains the student questionnaire in English. (DOCX)

S3 File This file contains the student questionnaire in Dari. (DOCX)
