# Supplementary material for: Children's peer violence perpetration and victimization: Prevalence and associated factors among school children in Afghanistan
Source: PLoS One. 2018 Feb 13;13(2):e0192768. doi: 10.1371/journal.pone.0192768 (PMC5811021; doi:10.1371/journal.pone.0192768)
Supplement: S3 File — (DOCX) [file pone.0192768.s003.docx]

فهرست **B:** سوالنامه شاگرد

ای دی نمبر شاگرد:.........................................

نام مکتب:.....................................................

ای دی نمبر مکتب:..........................................

2018 2017 2016 نوبت مصاحبه (حلقه نمایید):

تاریخ:..........................................................

| درباره شما | | |
| --- | --- | --- |
| .............. سال | چند سال دارید ؟ | 1 |
| 1. پسر 2. دختر | جنسیت؟ | 2 |
| 3 **صنف چند هستید؟** ________________________________ | | |
| ___________ تعداد | در فامیل شما چند نفر زندگی میکند؟ | 4 |
| ___________ تعداد | چند برادر دارید ؟ | 5 |
| ___________ تعداد | چند خواهر دارید ؟ | 6 |
| مکتب | | |
| 7در جریان 4 هفته گذشته چند روز به مکتب نیامده بودید؟ ؟ تعداد روز________ | | |
| - مریض بودید ۱- بلی ۲- نخیر - کرایه راه نداشتید ۱- بلی ۲- نخیر - بخاطر کارهای خانه ۱- بلی ۲- نخیر - کارکردن جهت دریافت پول ۱- بلی ۲- نخیر - ویا بخاطر کدام خشونت ۱- بلی ۲- نخیر - یا کدام دلیل دیگر ۱- بلی ۲- نخیر | آخرین باری که شما به مکتب نیامده بودید دلیل چی بود؟ | 8 |
| 1. هیچ نمیتواند بخواند 2. خوانده میتواند اما با بسیار مشکلات 3. میتواند بخواند اما سکته سکته 4. روان خوانده میتواند | شما چگونه میتوانید خوب بخوانید؟  از شاگر بخواهید که همین سوال را از روی پرسشنامه بخواند.این متن را خوانده می توانید؟  (یک خط را برای خواندن به شاگرد اینجا بنویسید) | 9 |
| 1. در تقسیم مشکل دارد 2. در جمع کردن مشکل دارد 3. به آسانی میتواند جمع کند 4. به آسانی میتوان تقیسم نماید | **در مضمون ریاضی چطور هستید** ؟  توسط مصاحبه کننده امتحان شود:  16+4 = 7+3 = 25 / 5 = | 10 |

| بلی، بسیار مشکل دارم | بلی، کمی مشکل دارم | گاه گاهی احساس میکنم | خیر، مشکلی ندارم |  |  |
| --- | --- | --- | --- | --- | --- |
| 4 | 3 | 2 | 1 | **شما کدام مشکل بینایی دارید؟** درصورت داشتن پرسیده شود.**حتی اگر عینک هم بپوشد** | 11 |
| 4 | 3 | 2 | 1 | **کدام مشکل شنوایی ؟** | 12 |
| 4 | 3 | 2 | 1 | **کدام مشکل پای؟ در راه رفتن، یا بالا و پایین شدن ؟** | 13 |
| 4 | 3 | 2 | 1 | **در حافظه، تمرکز و یادگیری چطور ؟** | 14 |
| 4 | 3 | 2 | 1 | بهتر است این سوال درصورت نداشتن مشکل تکلم شاگرد پرسیده نشود.  **آیا شما در صحبت کردن مشکل دارید؟** | 15 |

| شما چگونه احساس میکنید  حالا ما در مورد اینکه شما چگونه احساس میکنید سوالات را میپرسیم. من سه عبارات را برای تان میخوانم شما بگوئید که کدام آن حالت تانرا در دو هفته گذشته خوبتر بیان میکند. | | |  |
| --- | --- | --- | --- |
| **3** | **2** | **1** | **شماره** |
| من خفه نبودم. | من یک بار در یک زمان خفه بودم. | من اکثر اوقات خفه بودم. | 201a |
| همه چیز درست و به میل شما بود | مطمئن نیستید چیزی به میل شما بوده باشد | هیچ چیز به میل شما نبود | 201b |
| همه چیز را اشتباه مکردید | بعضی چیزها را اشتباه میکردید | خیلی چیزها را به خوبی پیش میبردید | 201c |
| اصلا هیچ چیز برای تان خوش آیند نبود | در بعضی موارد خوش بودید | دربسیاری موارد خوش بودید | 201d |
| در فامیل مهم نیستید | مطمئن نیستید که برای فامیل تان مهم باشد | برای فامیل تان مهم هستید | 201e |
| بلی خودت را دوست دارید | از خود راضی نیستید | از خودت نفرت داشتید | 201f |
| کارهای بد معمولآ اشتباه شما نبود | اکثرآ افعال بد اشتباه شما بود | همه افعال بد اشتباه شما بود | 201g |
| بلی خواستید که خود کشی بکنید | درباره خودکشی فکر میکنید اما این کار را نمی کنید | در باره خودکشی هیچ فکر نمیکنید | 201h |
| گاه گاهی احساس گریه برای تان دست می داد | چندین بار احساس گریه میکردید | هر روز احساس گریه برای تان دست میداد و یا هیچ نمی داد | 201i |
| هیچ وقت احساس آزاردگی نکردید | بعضی اوقات احساس می کردید | هردوهفته احساس آزرده گی داشتید | 201j |
| اصلا نخواستید با مردم باشید | بعضی اوقات دوست نداشتید با مردم باشید | دوست داشتید بامردم باشید | 201k |
| در مورد هرچیز خیلی به ساده گی تصمیم گرفته بودید | خیلی سخت بوده برای تان تصمیم گرفتن | نتوانسته اید چیزی را تصمیم بگیرید | 201l |
| بسیار بد قواره معلوم می شدید | بعضی چیزهای بد در قیاقه خود احساس می کردید | شما خوب معلوم میشدید | 201m |
| انجام کارهای مکتب کدام مشکل بزرگ برای تان نبوده | بعضی اوقات باید خود تان را بسیار مجبور کنید تا کارهای مکتب را تمام کنید | همیشه باید خود تان را مجبور کنید تا کارهای مکتب تان را انجام دهید. | 201n |
| هردو هفته خیلی راحت بودید | بعضی شب ها خواب راحت نداشتید | هر شب هنگام خواب ناراحت بودید | 201o |
| همیش خسته می بودید | بعضی روزها خسته میبودید | یگان باراحساس خسته گی میکردید | 201p |
| بسیار خوب غذا میخورید | بعضی روزها اشتهای خوردن نداشتید | بسیاری روزها اشتهای خوردن نداشتید | 201q |
| هردو هفته تشویش درد را میکردید | چندین بار تشویش درد را میکردید | شما تشویش درد را نمیکنید | 201r |
| هردو هفته احساس تنهایی میکردید | بعضی اوقات احساس تنهایی میکردید | احساس تنهایی نکرده اید | 201s |
| بودن در مکتب بسیاری اوقات برای تان خوشایند است | بودن در مکتب گاهی گاهی خوش آیند است | بودن در مکتب برای تان خوشایند نیست | 201t |
| هیچ دوست و رفیق ندارید | فقط چند تا دوست دارید و خوش دارید دوستان بیشتر داشته باشید | بلی دوستان زیاد دارید | 201u |
| در مضامین که بسیار خوب بودید خوب نیستید | در دروس مکتب مثل گذشته خوب نیستید | در دروس مکتب خوب استید | 201v |
| حال هم مثل اطفال دیگر خوب هستید | اگر بخواهید میتوانید مثل اطفال دیگر بهترین باشید | هرگز نمی توانید مثل اطفال دیگر خوب باشید | 201w |
| باور دارید کسی ترا دوست دارد | متیقن نیستید کسی ترا دوست داشته باشد | هیچ کس ترا دوست ندارد | 201x |
| همیش با دوستان تان جروبحث میکنید | بسیاری اوقات با دوستان تان جروبحث میکنید | با دوستانم خوش هستید و جروبحث نمی کنید | 201y |
| هرگز از طرف روز نمیخوابید | بسیاری اوقات از طرف روز میخوابید | همیشه از طرف روز میخوابید | 201z |
| خوراک تان خوب است | **بعضی روز ها هیچ سیر نمی کنید** | بسیاری روز ها سیر نمی کنید | 201aa |
| بسیار برای تان مشکل است | **به یاد آوردن چیزی کمی برای تان دشوار است** | به یاد آوردن برای تان آسان است | 201ab |

| **منازعه/ میان اطفال** | | | | | |
| --- | --- | --- | --- | --- | --- |
| چهار بار یا بیشتر از آن | دو یا سه بار | یک بار | هیچ | در ماه گذشته**، چند بار اطفال دیگر همرایتان جنجال کردند؟** | |
| 3 | 2 | 1 | 0 | در ماه گذشته، **آیا کسی شمارا به لقب بد صدا زده؟** | 301 |
| 3 | 2 | 1 | 0 | در ماه گذشته، **آیا کوشش کردند شما را با دوستان تان بیآندازند**؟ | 302 |
| 3 | 2 | 1 | 0 | در ماه گذشته**، آیا بدون اجازه چیزهای شما را گرفتند؟** | 303 |
| 3 | 2 | 1 | 0 | در ماه گذشته**، آیا کسی به قیافه شما تمسخر کرده؟** | 304 |
| 3 | 2 | 1 | 0 | در ماه گذشته**، کدام شوخی توهین آمیز و یا تمسخر اما بدون قیافه**؟ | 305 |
| 3 | 2 | 1 | 0 | در ماه گذشته**، کدام پای پچلک که بیافتید؟** | 306 |
| 3 | 2 | 1 | 0 | در ماه گذشته**، تیله کردن قصدی که بیآفتید؟** | 307 |
| 3 | 2 | 1 | 0 | در ماه گذشته**، به نوعی زخمی کرده باشد؟** | 308 |
| 3 | 2 | 1 | 0 | در ماه گذشته**، کسی شما را لت و کوب کرده که زخمی شده باشید؟** | 309 |
| 3 | 2 | 1 | 0 | در ماه گذشته**، چیزی مربوط شما را شکستانده باشد؟** | 310 |
| 3 | 2 | 1 | 0 | در ماه گذشته**، آیا کسی کوشش کرده که شاگردان دیگر را بخاطر مخالفت با شما تحریک کند؟** | 311 |
| 3 | 2 | 1 | 0 | در ماه گذشته**، کسی از شما چیزی را دزدیده ؟** | 312 |
| 3 | 2 | 1 | 0 | در ماه گذشته**، کسی از صحبت کردن با شما انکار کرده؟** | 313 |
| 3 | 2 | 1 | 0 | در ماه گذشته**، افراد دیگر را وادارساخته باشد که با شما صحبت نکنند؟** | 314 |
| 3 | 2 | 1 | 0 | در ماه گذشته**، کسی قصدآ چیزرا که مربوط شما بود تخریب کرده؟** | 315 |
| 3 | 2 | 1 | 0 | در ماه گذشته**، آیا کسی برایتان دشنام زده؟** | 316 |
| چهار بار یا بیشتر از آن | دو یا سه بار | یک بار | هیچ | در ماه گذشته**،** این اتفاقات را در کجا تجربه کردید؟ |  |
| 3 | 2 | 1 | 0 | **در صنف یا در مکتب ؟** | 317 |
| 3 | 2 | 1 | 0 | **بیرون از مکتب ؟** | 318 |
| 3 | 2 | 1 | 0 | **در کوچه؟** | 319 |
| 3 | 2 | 1 | 0 | **درخانه ؟** | 320 |

| چهار بار یا بیشتر از آن | دو یا سه بار | یک بار | هیچ | مجازات مکتب | |
| --- | --- | --- | --- | --- | --- |
| 3 | 2 | 1 | 0 | در ماه گذشته**، چندبار معلم شما را لت و کوب یا مجازات فزیکی کرده؟** | 321 |
| 3 | 2 | 1 | 0 | در ماه گذشته**، چندبار معلم شمارا گوش مالی داده؟** | 322 |
| 3 | 2 | 1 | 0 | در ماه گذشته**، چندبار معلم شما را روی میز و یا چوکی ایستاده کرده؟** | 323 |
| 3 | 2 | 1 | 0 | در ماه گذشته**، چند بار معلم شما را بخاطرجزا دادن دوانده باشد؟** | 324 |
| 3 | 2 | 1 | 0 | در ماه گذشته**، چند بار معلم شما را مجبور کرد تا در داخل یا بیرون از صنف به زانور راه بروید؟** | 325 |
| 3 | 2 | 1 | 0 | در ماه گذشته**، چند مرتبه معلم شما را با چوب یا چیز دیگرجزا داد؟** | 326 |
| 3 | 2 | 1 | 0 | **مجازات در خانه** | |
| 3 | 2 | 1 | 0 | در ماه گذشته**، چند بار والدین تان شما را یا مجازات فزیکی کرد(سیلی زدن، لت و کوب)**؟ | 327 |
| 3 | 2 | 1 | 0 | در ماه گذشته**، آیا به اندازه لت و کوب شدید که زخم برداشته باشید؟** | 328 |

| چهار بار یا بیشتر از آن | دو یا سه بار | یک بار | هیچ | **خشونت های فامیلی** | |
| --- | --- | --- | --- | --- | --- |
| 3 | 2 | 1 | 0 | در ماه گذشته**، چند بار پدر تان را دیدید که با کسی دیگر جنگ فیزیکی کند؟** | **329** |
| 3 | 2 | 1 | 0 | در ماه گذشته**، چند بار شنیدید یا دیدید که پدر تان مادرتانرا لت و کوب کرده باشد؟** | **330** |
| 3 | 2 | 1 | 0 | در ماه گذشته**، چندبار شنیدید یا دیدید که مادر تان توسط عضوء فامیل تان لت وکوب شده باشد؟** | **331** |
| 3 | 2 | 1 | 0 | در ماه گذشته**، آیا بخاطر نبود غذا در خانه، گرسنه به مکتب رفتید؟** | **332** |
| 3 | 2 | 1 | 0 | در ماه گذشته**، آیا به علت نبود غذا گرسنه خوابیدید؟** | **333** |

| منازعه/ میان اطفال | | | | | |
| --- | --- | --- | --- | --- | --- |
| چهار بار یا بیشتر از آن | دو یا سه بار | یک بار | هیچ | در چهار هفته گذشته چند بار... |  |
| 3 | 2 | 1 | 0 | در ماه گذشته**، طفل دیگری را به نام های بد صدا زدید؟** | 401 |
| 3 | 2 | 1 | 0 | در ماه گذشته**، کوشش کردید طفل دیگر را با دوستانش به مشکل مواجه سازید؟** | 402 |
| 3 | 2 | 1 | 0 | در ماه گذشته**، آیا وسایل و یا اجناس طفلی را بدون اجازه او گرفته اید که باعث رنجش او شده باشید؟** | 403 |
| 3 | 2 | 1 | 0 | در ماه گذشته**، طفل دیگری را بخاطرچهره ظاهرش مسخره کردید؟** | 404 |
| 3 | 2 | 1 | 0 | در ماه گذشته**، طفل دیگری را به دلیلی مسخره کردید؟** | 405 |
| 3 | 2 | 1 | 0 | در ماه گذشته**، طفل دیگری را بخاطر اینکه به زمین بیافتد پا پچلک دادید؟** | 406 |
| 3 | 2 | 1 | 0 | در ماه گذشته**، طفل دیگری را جهت افگارشدن تیله کردید؟** | 407 |
| 3 | 2 | 1 | 0 | در ماه گذشته**، طفل دیگری را فیزیکی آسیب رسانیدید ؟** | 408 |
| 3 | 2 | 1 | 0 | در ماه گذشته**، طفل دیگری را لت و کوب کردید که جراحت بردارد؟** | 409 |
| 3 | 2 | 1 | 0 | در ماه گذشته**، قصدآ جنس مربوط طفل دیگر را شکستاندید**؟ | 410 |
| 3 | 2 | 1 | 0 | در ماه گذشته**، کوشش کردید که یک طفل را با اطفال دیگر مخالفت بیاندازید؟** | 411 |
| 3 | 2 | 1 | 0 | در ماه گذشته**، آیابعضی چیزهای طفل دیگر را دزدیدید؟** | 412 |
| 3 | 2 | 1 | 0 | در ماه گذشته**، از صحبت کردن با طفل دیگر انکار کردید؟** | 413 |
| 3 | 2 | 1 | 0 | در ماه گذشته**، اطفال دیگر را وادارساختید با طفل دیگر صحبت نکند؟** | 414 |
| 3 | 2 | 1 | 0 | در ماه گذشته**، قصدآ جنس مربوط طفل دیگر را تخریب کردید؟** | 415 |
| 3 | 2 | 1 | 0 | در ماه گذشته**، به طفل دیگری دشنام زده اید؟** | 416 |
| مجازات طفل | | | | | |
| جدآ موافق | موافق | مخالف | جدآ مخالف | درجملات که برایتان تحریر می کنم 4 گزینه را باید انتخاب کنید.و نظرتان را بامن شریک سازید(موافق، مخالف، جدآموافق، جدآ مخالف) |  |
| 3 | 2 | 1 | 0 | **به نظر شما اگر طفل اطاعت والدینش را نکند، باید لت و کوب شود؟** شما چطور؟ | 417 |
| 3 | 2 | 1 | 0 | **به نظر شما اگر اطفال جنگ کنند والدین باید آنها را لت و کوب کنند** | 418 |
| 3 | 2 | 1 | 0 | **به نظر شما اگر اطفال به گفت والدینشان نمی کند باید جزا لت و کوب برایشان داده شود** | 419 |
| 3 | 2 | 1 | 0 | **به نظر شما اگر یک طفل در مکتب بی ادبی میکند باید لت و کوب شود** | 420 |
| 3 | 2 | 1 | 0 | **شما فکر میکنید اگر طفل به شما صدمه بزند باید بالعکس جوابش را بدهید** | 421 |

| جایگاه خانم ها | | | | | |
| --- | --- | --- | --- | --- | --- |
| درقسمت اناث میخواهم که نظر بدهید که آیا شما با نظریات من، جداّ موافق، موافق، مخالف و یا جداّ مخالف هستید | | | | | |
| جدآ موافق | موافق | مخالف | جدآ مخالف | **به نظر شما دختران در فامیل تان باید مکتب بروند** | 501 |
| 3 | 2 | 1 | 0 | **به نظر شما مرد های فامیل تان باید به خانم های شان جهت رفتن به کلینیک اجازه بدهند** | 502 |
| 3 | 2 | 1 | 0 | **به نظر شما در فامیل شما مرد ها باید به نظریات خانم های شان در باره مکتب رفتن گوش دهند** | 503 |
| 3 | 2 | 1 | 0 | **به نظر شما خانم ها هم بخاطر مصارف در خانه باید نظر بدهند** | 504 |
| 3 | 2 | 1 | 0 | **به نظر شما خانمها باید بتوانند از علماء دین موضوعات دینی را پرسان کنند** | 505 |
| 3 | 2 | 1 | 0 | **به نظر شما شوهران باید به نظریات خانمهای شان در رابطه به موضوعات عایداتی گوش دهند** | 506 |
| 3 | 2 | 1 | 0 | **به نظر شما شوهر باید در فامیل مهربان و به خانم خود توجه بیشتر داشته باشد** | 507 |
| 3 | 2 | 1 | 0 | **به نظر شما خانم ها در فامیل همیش باید از شوهران خویش اطاعت کند** | 508 |
| 3 | 2 | 1 | 0 | **به نظر شما اگر خانم کار اشتباه کند شوهر حق دارد وی را مجازات کند** | 509 |
| 3 | 2 | 1 | 0 | **به نظر شما خانم ها باید در محافل خوشی وعروسی ها شرکت کنند** | 510 |
| 3 | 2 | 1 | 0 | **به نظر شما خانم ها باید در محافل اجتماعی همسایه ها نیز شرکت کنند** | 511 |
| 3 | 2 | 1 | 0 | **به نظر شما خانم ها باید در پروگرامهای آموزشی کسب مهارت شرکت کنند** | 512 |
| 3 | 2 | 1 | 0 | **به نظر شما خانم هاحق دارند درفعالیت های عایداتی شرکت کنند** | 513 |

| **سوالات ذیل سوالات نهایی است که ما میخواهیم شما نظر تان را ابراز نمایید** | | | | | |
| --- | --- | --- | --- | --- | --- |
| جدآ موافق | موافق | مخالف | جدآ مخالف |  |  |
| 3 | 2 | 1 | 0 | شما می توانید بخاطر بیرون شدن از یک وضعیت دشوار راه های زیادی را فکر کنید. | 514 |
| 3 | 2 | 1 | 0 | شما انرژی زیاد بخاطر تعقیب اهداف خود به خرچ میدهید | 515 |
| 3 | 2 | 1 | 0 | به نظر شما هر مشکل راه حل دارد | 516 |
| 3 | 2 | 1 | 0 | شما میتوانید بخاطر بدست آوردن چیزیکه در زندگی تان بسیار مهم است فکر کنید و چند راه پیدا کنید. | 517 |
| 3 | 2 | 1 | 0 | به نظر شما حتی در وضعیت که دیگران نا امید شوند، شما میدانید و میتوانید برای حل مشکل یک راهی را دریافت کنید | 518 |
| 3 | 2 | 1 | 0 | شما اهداف را که برای خود تعیین میکنید بدست میآورید | 519 |

تشکر از اینکه ما را کمک کردید،کمک شما سبب می شود تا اطفال دیگر را کمک نماییم.
